# Elevated Levels of SOX10 in Serum from Vitiligo and Melanoma Patients, Analyzed by Proximity Ligation Assay

**DOI:** 10.1371/journal.pone.0154214

**Published:** 2016-04-25

**Authors:** Andries Blokzijl, Lei E. Chen, Sigrun M. Gustafsdottir, Jimmy Vuu, Gustav Ullenhag, Olle Kämpe, Ulf Landegren, Masood Kamali-Moghaddam, Håkan Hedstrand

**Affiliations:** 1 Dept. of Immunology, Genetics and Pathology, Science for Life Laboratory, Uppsala University, Box 815, SE-751 08 Uppsala, Sweden; 2 Dept. of Radiology, Oncology and Radiation Science, Uppsala University, Uppsala, Sweden; 3 Dept. of Medical Sciences, Uppsala University, SE-751 08 Uppsala, Sweden; 4 Ludwig Institute for Cancer Research, Science for Life Laboratory, Uppsala University, Box 595, SE-751 24, Uppsala, Sweden; University of Alabama at Birmingham, UNITED STATES

## Abstract

**Background:**

The diagnosis of malignant melanoma currently relies on clinical inspection of the skin surface and on the histopathological status of the excised tumor. The serum marker S100B is used for prognostic estimates at later stages of the disease, but analyses are marred by false positives and inadequate sensitivity in predicting relapsing disorder.

**Objectives:**

To investigate SOX10 as a potential biomarker for melanoma and vitiligo.

**Methods:**

In this study we have applied proximity ligation assay (PLA) to detect the transcription factor SOX10 as a possible serum marker for melanoma. We studied a cohort of 110 melanoma patients. We further investigated a second cohort of 85 patients with vitiligo, which is a disease that also affects melanocytes.

**Results:**

The specificity of the SOX10 assay in serum was high, with only 1% of healthy blood donors being positive. In contrast, elevated serum SOX10 was found with high frequency among vitiligo and melanoma patients. In patients with metastases, lack of SOX10 detection was associated with treatment benefit. In two responding patients, a change from SOX10 positivity to undetectable levels was seen before the response was evident clinically.

**Conclusions:**

We show for the first time that SOX10 represents a promising new serum melanoma marker for detection of early stage disease, complementing the established S100B marker. Our findings imply that SOX10 can be used to monitor responses to treatment and to assess if the treatment is of benefit at stages earlier than what is possible radiologically.

## Introduction

Malignant melanoma (MM) is a life threatening disease with increasing incidence worldwide. Currently, few clinically useful serological markers are available, and prognosis is made by pathologists through histological evaluations of the excised primary tumor and clinical signs of metastases.

Clinically, the S100B protein is the most commonly used serum marker in MM patients for monitoring tumor responses to treatments in later stages and in recurrent disease [1]. There are no clinically established serological markers for melanoma detection at earlier stages. Assays for S100B are prone to false positive results, and levels of the marker correlate poorly with prognosis.

The transcription factor SOX10 is important for normal development and function in melanocytes and nerve cells [2], and it has been demonstrated to promote the development of giant congenital naevi and melanomas [3].

SOX10 is a member of the HMG (high-mobility group) box superfamily, belonging to the E subgroup within the SOX family. SOX10 has been reported to be essential for neural crest cell fate decisions [2]. Mutations in the *sox10* gene are associated with the Waardenburg-Shah syndrome and Hirschprung´s disease [4–6]. SOX10 also has important roles in the normal development of melanocytes [7] and take parts in the regulation of Microphthalmia Transcription Factor (MITF) which is important for melanogenesis[8]. Expression of SOX10 mRNA [9] and protein has also been reported in other normal tissues [10]. In tumors, SOX10 expression has been detected in most MMs and metastases [11], gliomas [12], malignant peripheral nerve sheath tumors [10], clear cell sarcoma [13], invasive breast carcinomas [14] and salivary adenoid cystic carcinomas [15].

In this work, we describe the detection of SOX10 in blood serum from vitiligo and melanoma patients. Mechanisms behind the development of vitiligo have been debated [16] and may include a disturbed regulation of local and global homeostasis by the skin's neuroendocrine system [17]. In autoimmune disorders such as vitiligo, an immune response against melanocytes mediates tissue destruction. In the autoimmune response during vitiligo development, both humoral and cellular mechanisms are believed to be involved in cytotoxicity [18, 19]. Additionally, these immune mechanisms have been demonstrated in vitiligo associated with MM [20]. Therefore, we hypothesized that such melanocyte destruction potentially released SOX10, an intracellular, non-secreted protein, to the circulation by cell lysis.

We investigated if SOX10 could be detected in serum using a sensitive proximity ligation assay (PLA) [21]. The assay requires three independent recognition events towards three separate epitopes on the SOX10 protein, thus substantially reducing the possibility of unspecific detection of other proteins. Besides overexpressed in malignant melanoma, SOX10 has also been reported to be expressed in other tumors of neural crest origin[22], such as neurofibroma. This study, where Proximity ligation assay is applied to detect SOX10 in serum for the first time is focus on melanoma patients. We therefore excluded patients with other malignancies, thus none of our patients would be expected to have SOX10 release from another SOX10 over expressing tumor.

We detected markedly elevated levels of SOX10 in the circulation of vitiligo patients. The finding prompted us to investigate if SOX10 could also be detected in sera from patients with MM. We collected serum samples from MM patients from two clinical institutions in Sweden. In total, we investigated the levels of SOX10 in 195 patients, 85 with vitiligo and 110 with MM, and compared the levels to those of 85 healthy controls, demonstrating the added value of SOX10 measurement in MM.

The aim of this study was to investigate if SOX10 could be detected with proximity ligation assay in serum. If any correlations to clinical findings in melanoma patients could be seen, further studies will be needed to further clarify statistical significance of the SOX10 level variations between melanoma subgroups found in this study.

## Material and Methods

### Vitiligo sera

Sera from patients with vitiligo at the outpatient ward of the Dermatology clinic, Uppsala University Hospital, were collected with approval from Regional ethics review board in Uppsala (ref. nr UPS 02–415) between September 2005 and December 2008. Sera were included and analyzed from 85 patients, 49 women and 36 men.

### Melanoma sera

#### Uppsala MM patients

Patients signed an informed consent form, approved by Regional ethics review board in Uppsala (ref. no 2010/386). Sera were collected at the Oncology clinic at Uppsala University Hospital. Most patients were enrolled in the routine control program after primary tumor excision during the period March 2011- January 2013. Altogether 119 patients were included. In the end, results from serum analyses in duplicates were achieved in 148 serum samples from 110 patients. Of these patients, 22 had two or more samples analyzed, while for the rest a single sample was analyzed. For one of the patients, tumor growth was too deep to be measured, and two patients were initially admitted to the clinic with metastases from a primary tumor of unknown location. These three patients were considered as high-risk patients. Two other patients had a primary eye MM. At inclusion there were 11 patients with *in situ* MM, 46 with MM in stage I, 18 in stage II, 23 in stage III and 12 in stage IV. There were four patients that shifted to stage IV during follow up; one from stage I, two from stage II, and one from stage III. Mean age at diagnosis was 62 years (23–92 years, median 65). 62 were women and 48 men. Eight patients received treatment. Four patients were included in the adjuvant DERMA study, a Phase III randomized, blinded, placebo-controlled immunization trial with a MAGE-A3 cancer antigen. Tree patients with advanced disease were included in the METRIC study, receiving trametinib or chemotherapy [23]. One patient with metastases received vemurafenib, a B-Raf enzyme inhibitor.

#### Gävle MM patients

In order to obtain sera from MM patients before the primary tumor was excised, three patients were included from the Dermatology clinic of Gävle Hospital. Two of these three patients later showed to have thin superficial spreading melanoma and one had an *in situ* melanoma.

### Control sera

Sera from healthy blood donors was collected at Uppsala University Hospital, and altogether 45 sera from normal individuals, matched with the patients of the Uppsala MM cohort for age and gender, were used as controls in the MM part of this study. Forty unmatched control sera were used for the vitiligo part of the study that was collected from healthy blood donors at the Uppsala University Hospital.

### Sampling and storage of serum

The sera were aliquoted and stored in -70°C before PLA analyses.

### Preparation of PLA probes

The polyclonal SOX10 antibody AF2864 was purchased from R&D Systems. The antibody recognizes the N-terminal part of SOX10, outside the conserved HMG box. PLA probes were constructed by covalently attaching two different oligonucleotides (SLC1 and SLC2) to two aliquots from the same batch of the polyclonal antibodies. The sequences for all the oligonucleotides used in the PLA are listed in S1 Table. Conjugation was performed as previously described [24] with the minor modification that the buffer used for the reduction of oligonucleotides was 1xPBS pH 7.4 with 5 mM EDTA.

### Capture beads preparation

An aliquot from the polyclonal SOX10 antibodies was used to couple to the Dynabeads M-270 Epoxy magnetic beads (Invitrogen, WI, USA). For the antibody coupling, 3 μg antibodies were coupled to 1 mg of the dry magnetic beads. The subsequent treatment and storage of the beads followed the protocol recommended by the manufacturer.

### Solid-phase proximity ligation assay

For each reaction the storage buffer of 1 μL of antibody-coated magnetic beads was replaced by 45 μL of PLA buffer (0.1% BSA, 100 nM goat IgG, 1 mM Biotin, 100 mg/mL ssDNA, 4 mM EDTA, 1X PBS, 0.05% Tween 20), and mixed with 5 μL patient serum samples. During the antibody capture step, reactions were incubated for 1.5 hr at room temperature (RT) under rotation. The magnetic beads were washed twice with wash buffer (1xPBS, 0.05% Tween 20), and 50 μL of the pair of PLA probes at a final concentration of 250 pM each was added to each well. The second incubation was carried out for 1.5 hr at RT under rotation, followed by two washes. Finally, 50 μL of ligation/PCR mix (1X PCR buffer (Quanta Biosciences, Gaithersburg, US), 25 mM MgCl_2_ (Quanta Biosciences), 0.1 μM of each primer Biofwd and Biorev, 0.1 μM TaqMan probe, 0.08 mM ATP, 100 nM connector oligonucleotide, 0.2 mM dNTPs, containing dUTP in place of dTTP. (Fermentas, Thermo scientific, Waltham, US), 1.5 units AccuStart Taq polymerase (Quanta Biosciences), 0.5 units T4 DNA ligase (Fermantas), 0.1units uracil-DNA glycosylase (Fermantas)) were added, followed by a 5 min incubation at RT for the ligation step before qPCR was performed on a Mx-3000 qPCR instrument (Stratagene, La Jolla, US), with an initial incubation for 4 min at 95°C, followed by 40 cycles of 15 s at 95°C and 1 min at 60°C. For non-template control (NTC) the PLA reaction was performed omitting sera.

### Data analyses

The Ct value was automatically calculated by Mxpro software (Stratagene) and the recorded Ct value was exported and further analyzed by Microsoft Excel software. For statistical analyses the 1-tail two sample heteroscedastic T-test was applied.

### S100B analyses

Patient sera were analyzed at the clinical laboratory at Uppsala University Hospital, using an automated chemistry analyzer instrument (Cobas E, Roche, Mannheim, Germany). The Cobas instrument is applied for routine analyses of S100B levels at the clinical chemistry unit, Uppsala University Hospital.

## Results

### SOX10 PLA analyses of patient samples

**Fig 1:**
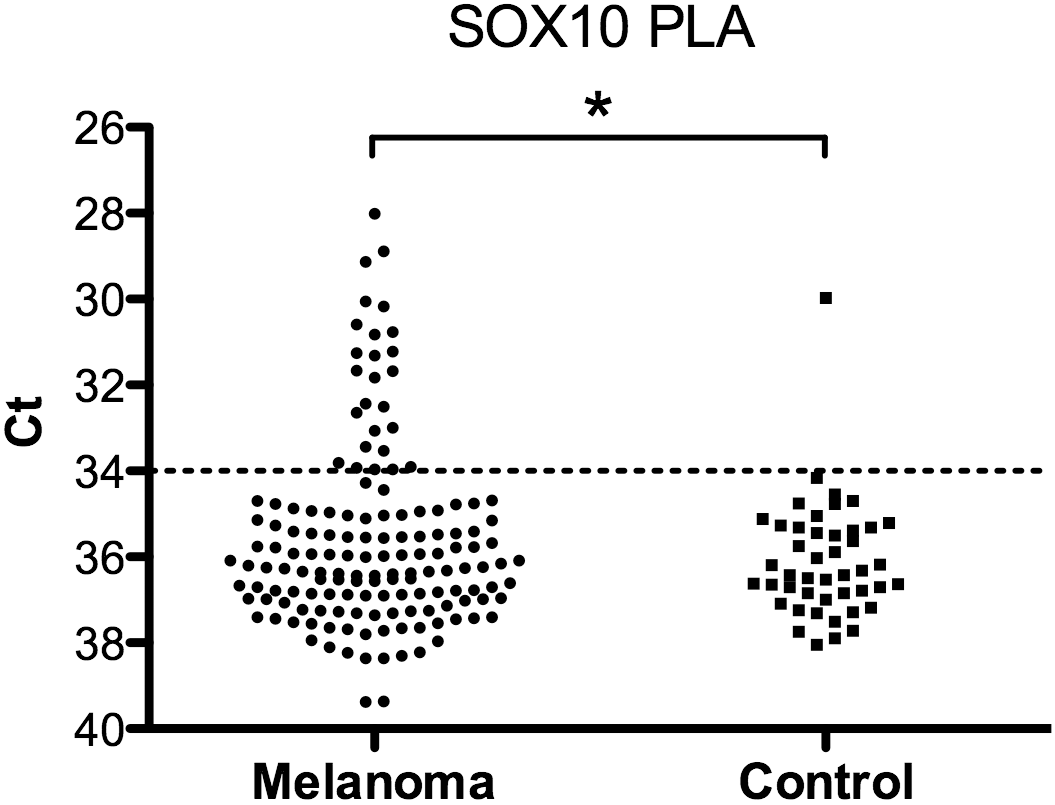
Measurement of SOX10 via solid-phase PLA in sera from MM patients. Ct values in sera of 110 patients (in total 148 MM samples) (left) and 45 control sera samples (blood donors) (right). For statistical analyses 1-tail two sample heteroscedastic t-test was applied. * P< 0.05. Line indicates cut-off for samples calculated as positive.

Normal levels of SOX10 protein in serum are expected to be very low. We did not detect SOX10 in healthy blood donors; except for in a serum sample of one donor (Fig 1). We considered samples as positive where all reactions showing a cycle threshold (Ct) value in the qPCR analysis of the PLA reactions lower than those of the healthy control with the lowest values. Lower Ct values correspond to higher SOX10 concentrations, as the Ct level is inversely correlated to the logarithm of the concentration of the target template. The Ct defines the number of cycles required for the fluorescent signal to exceed a preset threshold. In the PLA reaction, the antigen (SOX10) is first captured by an antibody immobilized on beads. After washes, two antibody preparations, each conjugated with a unique oligonucleotide, are added, followed after a new incubation by renewed washes. Oligonucleotide pairs, brought in proximity upon binding to a target protein by the antibodies they are attached to, are next ligated by the assistance of a connector oligonucleotide, and the ligation products then serve as templates for qPCR assays. Thus, the reaction requires recognition of three independent epitopes on the target protein in order to generate a signal. To be scored as positive, both runs within a technical duplicate for each individual patient serum sample were required to have a Ct value lower than the mean Ct values of the lowest control sample, in each test run. In order for a serum sample to be scored as positive, it had to be positive in at least two independent measurements. These criteria are equivalent to the criteria we used in previous work [25]. Using these criteria, only one (1.2%) out of the sera from 85 control individuals was consistently found to be positive for SOX10.

### Uppsala MM patients

In the Uppsala cohort of MM patients, we observed elevated serum SOX10 concentrations, compared to sera from healthy blood donors (Fig 1). The clinical data is presented in Tables 1–4. In total, 21 (19%) out of 110 MM patients were SOX10 positive, two (18%) of the 11 with *in situ* MM, seven (15%) among 46 low-risk MM patients (Stage I) and 12 (23%) among 53 middle-high risk patients (Stage II-IV) as shown in Table 1. One low-risk patient progressed with metastases. Results for all 54 patients with stage II-IV MM are presented in Table 3.

**Table 1:** Stages when 21 MM patients were found to be SOX10 positive.

Stage	TIS^1^	I (T1)	I (T2)	II	III-IV
SOX10 positive	2(18%)	6(22%)	1(5%)	1(6%)	11(31%)
Total	n = 11	n = 27	n = 19	n = 18	n = 35

^1^TIS, Tumor *in situ*.

**Table 2:** PLA-SOX10 vs. Cobas-s100 in 72 MM patients.

Stage	TIS^1^	I (T1)	I (T2)	II	III-IV
SOX10 positive	2(20%)	4(24%)	0	1(11%)	9(35%)
S100 positive	1(10%)	2(12%)	0	2(22%)	8(29%)
SOX10 and S100 positive	0	1(6%)	0	0	2(8%)
Total	n = 10	n = 17	n = 9	n = 9	n = 27

^1^TIS, Tumo*r in situ*.

**Table 3:** SOX10 reactivity in 54 middle-high-risk MM patients (stage II-IV).

Total positive 12		Total negative 42		
		recent relapse 25		no relapse 17
positive before or at relapse	7	regression or treatment response	12	
positive just after treatment started, then negative	2	lymph- or regional metastases	10	
continuously positive, yet no recurrent disease	3	internal progressive disease	3	

**Table 4:** SOX10 reactivity over time in nine patients during treatment follow up. DERMA and METRIC study treatment regimens are described under Methods.

Patient No.	Treatment	Clinic	SOX10 reactivity	No samples
1	DERMA	No recurrence	positive initially at first treatment dose, then negative during treatment	7
2	Vemurafenib	Regression	negative	3
3	DERMA	Lymph met	negative	3
4	METRIC	Regression	Initially negative, then positive. Recurrence 2 months later	2
5	DERMA	Recurrence	Initially negative, positive 10 months before recurrence. Develops vitiligo.	5
6	Post surgery	Regression	initially positive SOX10, then negative with lung hilus tumor regression	5
7	METRIC	Regression	positive, progress 2 months later	1
8	DERMA	No recurrence	negative	2
9	METRIC	Regression	negative	3

### Positive stage II-IV MM patients

Nine (75%) out of the 12 (22%) positive stage II-IV patients showed a positive SOX10 value closely correlated to tumor recurrence. Seven patients were positive before or at relapse, and two were positive just after treatment had started, with later samples being negative.

### Negative stage II-IV MM patients

Of the 42 (78%) stage II-IV patients with negative SOX10, 17 had not yet any relapse but 25 had a relapse recently or earlier on. Of these 25 patients, 12 (48%) were under pharmaceutical treatment with signs of treatment response, or had a single known metastasis already removed, thus they were in a “recurrence free” period. The remaining 13 (52%) patients had progressive disease, of which 10 presented with local or lymph node metastases, and three with disseminated internal disease.

### Patients under treatment

In total, 38 patients had metastases (stage III-IV) and nine (24%) were receiving medical or surgical treatment during the follow-up period. The treatment regimens, clinical outcome, SOX10 reactivity, and number of serum samples analyzed during the observation period are listed in Table 4. The clinical state was assessed by an oncologist through a pathology report, based on clinical and radiological examination. In patient No. 3 a lymph node metastasis appeared during treatment with no increased SOX10 reactivity in serum. In three patients SOX10 was negative with stable or regressive disease (patients No. 2, 8 and 9). SOX10 turned from positive to negative in patient No. 1 as treatment started, and also in patient No. 6 as signs of spontaneous tumor regression were seen on CT scans. In patients No. 4, 5 and 7 SOX10 turned from negative to positive several months ahead of clinical recurrence, although two patients initially showed clinical signs of regression. Patient No. 5 developed focal vitiligo during treatment.

### Gävle MM patients

We also analyzed samples collected from three patients with newly diagnosed MM visiting the dermatology clinic of Gävle. These samples were collected when the tumor was still confined to the skin. Of these, two had superficially spreading MM and for these SOX10 was positive. The third patient had an *in situ* MM and the serum sample was negative for SOX10.

### Vitiligo patients

**Fig 2:**
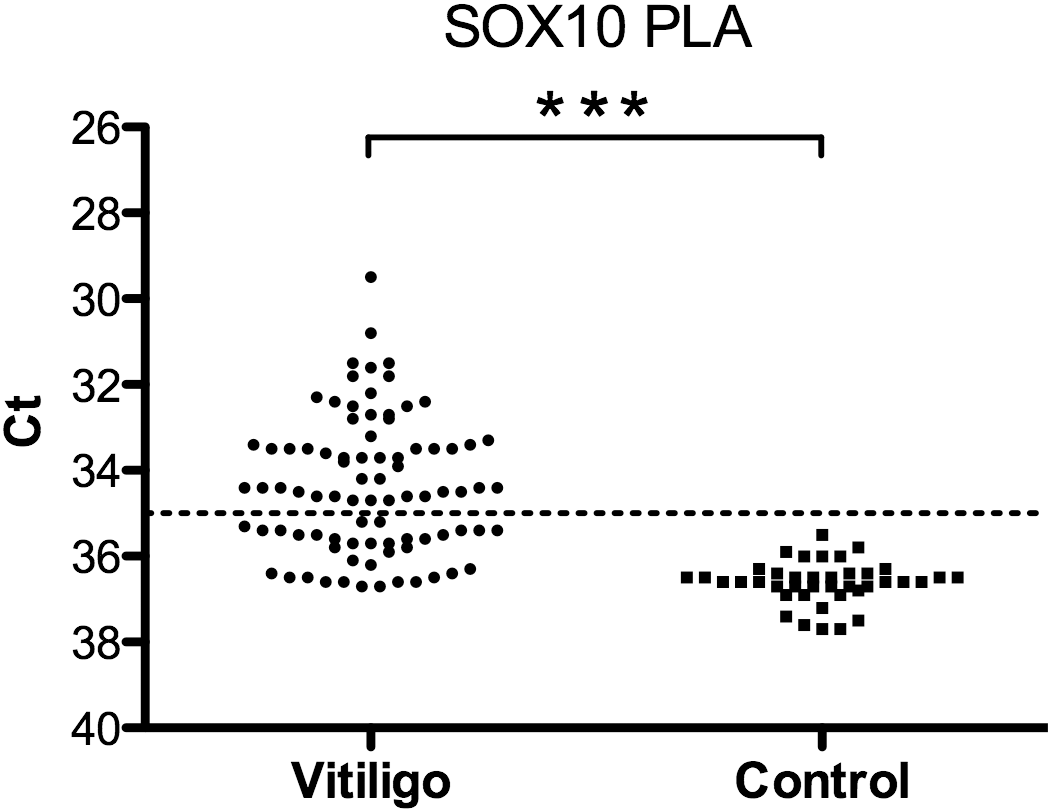
Measurement of SOX10 via solid-phase PLA in sera from vitiligo patients (n = 85) and from controls (healthy blood donors) (n = 40). Mean Ct (y-axis) values of duplicate samples are shown. For statistical analyses 1-tail two sample heteroscedastic t-test was applied. *** P<0.001. Line indicates cut-off for samples calculated as positive.

For the vitiligo cohort (Fig 2) we observed increased levels of SOX10 in 51 (60%) out of the 85 analyzed sera.

### PLA-SOX10 compared to Cobas-S100B

We compared the SOX10 measurements by PLA with those of S100B by Cobas Analyzer as routinely used in the clinic. For 72 MM patients Cobas-S100B and PLA-SOX10 were analyzed on the same occasion. We detected elevated levels of both SOX10 and S100B, both in groups with thin melanoma *in situ* and in later stages of the disease.

## Discussion

We demonstrate for the first time that detection of the transcription factor SOX10 is possible in serum via a highly specific and sensitive PLA. We present data indicating a promising role for SOX10 as a new serum marker in MM. Furthermore, we detected elevated SOX10 levels in vitiligo patients, suggesting that SOX10 is released from melanocytes by similar mechanisms in vitiligo and MM.

SOX10 was chosen as a marker in this study since the expression of this transcription factor is restricted to a few tissues, mainly melanocytes [7] and nerve cells [4–6]. We have previously compared the detection limits of PLA- and ELISA-based detection assays [25]. The study demonstrated the capacity of PLA to detect lower amounts of several proteins compared to state-of the art sandwich ELISA. For the transcription factor SOX10, the level in circulation is expected to be very low. In this study, only 1% of the serum samples from control subjects exceeded a set threshold with the assay format we present here. However, in vitiligo and MM sera, SOX10 was found in much higher frequencies. This increased release of SOX10 to the blood may reflect an underlying destruction and/or increased turnover of SOX10-containing cells in these conditions. A possible common mechanism is T-cell mediated destruction of melanocytes[26]

Further, in MM, we found SOX10 positive patients in both the low-risk group and the groups with high-risk and advanced disease. Interestingly, SOX10 was detected in fewer patients with thicker low-risk (T2A) melanomas or non-spread high-risk (stage II) MM. For these SOX10 positive patients, showing no sign of recurrent disease, a possible explanation for the SOX10 release could be functional immune surveillance and lysis of melanoma cells by immune mechanisms.

We also found some high-risk patients with advanced disease without elevated SOX10 levels. These 13 SOX10 negative patients, that had so far untreated metastatic disease, may represent an immunosuppressed subgroup[27], or a subgroup that express no or very low levels of SOX10 in their tumors, as we have reported previously [11]. In that report we found that lymph node metastases expressed significantly lower SOX10 levels than primary tumors or non-lymph node metastases. Interestingly, in line with our results, studies by others showed no correlation with preoperative values of S100B in serum and histopathological signs of metastases in sentinel lymph nodes [28].

In a subset of our patients we were able to follow SOX10 levels over time. We found that in 9 (75%) of the 12 positive stage II-IV MM patients detection of SOX10 in serum was related to relapse. Moreover, 12 (48%) of the 25 SOX10 negative patients, with recent relapse were under treatment, and remained negative. Nine patients were receiving medical or surgical treatment. A lymph node progression in one of these patients did not give rise to raised SOX10 but for the remaining eight (89%) patients SOX10 levels corresponded to a clinical treatment response. In these cases SOX10 remained negative in the patients who were responding to treatment and turned positive in the patients with signs of recurrence. In two of the responding patients, the SOX10 reactivity was furthermore shown to return to normal levels soon after treatment was started. The observations indicate that SOX10 could serve as a dynamic marker for monitoring treatment responses and has the potential to assess clinical benefit earlier than radiological examinations.

Previous studies have suggested that cell damage and necrosis are the main sources of S100B in serum [29]. Our finding of SOX10 in blood indicates on-going destruction of SOX10-containing cells, possibly through immune-mediated mechanisms. As is the case for vitiligo, signs of immune-related depigmentation have also been associated with MM. These signs include regression phenomena, halo nevi and melanoma-associated depigmentation [30]. Furthermore, the vitiligo-like lesions seen in melanoma-associated depigmentation have frequently been reported to be associated with a better prognosis [31, 32]. A possible explanation for the SOX10 positive subgroup of low-risk patients seen here is an immune response against the primary tumor at an earlier stage in this subgroup of patients. In other patients, the lack of release of SOX10 from metastases in lymph nodes and in transit metastases, may be consequences of a suppressed cellular immune system[27].

The recent breakthrough of biologics against immune checkpoint inhibitors such as PD1 and CTLA-4 in melanoma ([33]) has demonstrated unprecedented clinical responses due to reduced immune suppression. Interestingly, the discovery of elevated SOX10 levels in vitiligo patients suggests that increased immune mediated lysis of melanoma cells has the potential to increase serum concentrations of SOX10. Future studies are needed to investigate if SOX10 has the potential to aid in treatment selections of immune check point inhibitors. In this study we demonstrate for the first time that SOX10 is measurable in serum. Our discovery suggests that SOX10 can be used to monitor the response to treatment and to assess if the treatment is of benefit at earlier time points before radiologically evident. Further studies are warranted to confirm these results and to investigate if measurement of SOX10 has prognostic value.

Future biomarker use for melanoma diagnosis will likely be based on a combination of markers for improved accuracy. The discovery of SOX10 in the serum of melanoma patients suggest that SOX10 could be included in larger combinatorial biomarker panels.

By using a novel SOX10 proximity ligation assay we demonstrate in this study for the first time that elevated levels of SOX10 is detected in the serum from melanoma patients.

## Supporting Information

S1 Table Oligonucleotide sequences in Sox10 PLA assay. (DOCX)
