# Supplementary material for: Elevated Levels of SOX10 in Serum from Vitiligo and Melanoma Patients, Analyzed by Proximity Ligation Assay
Source: PLoS One. 2016 Apr 25;11(4):e0154214. doi: 10.1371/journal.pone.0154214 (PMC4844164; doi:10.1371/journal.pone.0154214)
Supplement: S1 Table — (DOCX) [file pone.0154214.s001.docx]

Supplemental Table 1.

**Oligonucleotide sequences in Sox10 PLA assay**

| Name | Sequence | Modification | Company |
| --- | --- | --- | --- |
| SLC1 | CGCATCGCCCTTGGACTACGACTGACGAACCGCTTTGCCTGACTGATCGCTAAATCGTG | 5’-Amine | Integrated DNA Technology |
| SLC2 | TCGTGTCTAAAGTCCGTTACCTTGATTCCCCTAACCCTCTTGAAAAATTCGGCATCGGTGA | 3’-Amine, 5’-Phosphate | Integrated DNA Technology |
| Biofwd | CATCGCCCTTGGACTACGA |  | Integrated DNA Technology |
| Biorev | GGGAATCAAGGTAACGGACTTTAG |  | Integrated DNA Technology |
| TaqMan probe | TGACGAACCGCTTTGCCTGACTGA | 5’-FAM, 3’-MGB | Applied Biosystems |
